# Cyclin D1 G870A Polymorphism and Risk of Nasopharyngeal Carcinoma: A Case-Control Study and Meta-Analysis

**DOI:** 10.1371/journal.pone.0113299

**Published:** 2014-11-19

**Authors:** Dan Liao, Yongfu Wu, Xingxiang Pu, Hua Chen, Shengqun Luo, BinBin Li, Congcong Ding, Guo-Liang Huang, Zhiwei He

**Affiliations:** 1 Sino-American Cancer Research Institute, Guangdong Medical College, Dongguan, China, and Key Laboratory for Medical Molecular Diagnostics of Guangdong Province, Dongguan, China; 2 Department of Medical Oncology, Hunan Tumor Hospital, Changsha, China; Gentofte University Hospital, Denmark

## Abstract

**Background:**

Cyclin D1 (CCND1) plays a key role in cell cycle regulation. It is a well-established human oncogene which is frequently amplified or overexpressed in cancers. The association between CCND1 G870A polymorphism and cancer risk has been widely assessed. However, a definitive conclusion between CCND1 G870A polymorphism and risk of nasopharyngeal carcinoma (NPC) remains elusive.

**Methods:**

We firstly performed a hospital-based case-control study involving 165 NPC cases and 191 cancer-free controls in central-south China, and then conducted a meta-analysis with six case-control studies to evaluate the association between NPC risk and CCND1 G870A polymorphism.

**Results:**

The case-control study found a significant association between CCND1 G870A polymorphism and NPC risk in various comparison models (AA vs. GG: OR = 2.300, 95% CI 1.089–4.857, p = 0.029; AG vs. GG: OR = 2.832, 95% CI 1.367–5.867, p = 0.005; AA/AG vs. GG: OR = 2.597, 95% CI 1.288–5.237, p = 0.008; AA vs. AG/GG: OR = 0.984, 95% CI 0.638–1.518, p = 0.944). Further meta-analysis showed that there was no significant association between CCND1 G870A polymorphism and NPC risk in overall analysis. In the stratified analysis by race, however, significant associations were only found in Caucasians (for the allele model A vs. G: OR = 0.75, 95% CI 0.59–0.97, p = 0.03; for the co-dominant model AA vs. GG: OR = 0.52, 95% CI 0.32–0.86, p = 0.01; for the dominant model AA/AG vs. GG: OR = 0.49, 95% CI 0.32–0.74, p<0.01; for the recessive model AA vs. AG/GG: OR = 0.90, 95% CI 0.61–1.34, p = 0.60).

**Conclusions:**

A significant association between CCND1 G870A polymorphism and NPC risk was found in the central-southern Chinese population. The meta-analysis indicated that CCND1 G870A polymorphism may contribute to the development of NPC in Caucasians.

## Introduction

Nasopharyngeal carcinoma (NPC) is considered as one of the rarer cancer forms globally and has a significantly different ethnic and geographic distribution [1]. The GLOBOCAN 2012 report from the World Health Organization’s International Agency for Research on Cancer (IARC) shows that there are nearly 86691 new cases and 50828 deaths die of NPC in 2012 [2]. The etiology of NPC is thought to be tied with a complex interaction of genetic, viral, environmental and dietary factors [3]. However, genes involved in the development of NPC are still unclear.

The abnormality of G_1_-S phase progression of the cell cycle is one of the hallmarks of cancer. The checkpoint of G_1_/S is frequently altered in many epithelial tumors and may confer growth advantage and increased tumorigenesis [4]. The G_1_ transition to S phase is regulated by cyclins, cyclin-dependent kinases and their inhibitors [5]. Cyclin D1 (CCND1), which located on chromosome 11q13, is a crucial junction at the G_1_/S checkpoint of the cell cycle. CCND1 is a well-established human oncogene. It is frequently amplified or overexpressed in cancers, such as breast cancer, lung cancer, colorectal cancer, melanoma and oral squamous cell carcinomas, through copy number alterations, mutation, or as a consequence of the deregulation of mitogenic signalling downstream of oncogenes such as ERBB2 [6].

Owing to the important of cyclin D1 in the cell division, CCND1 single nucleotide polymorphism (SNP) has been investigated in a variety of tumors, such as uterus, breast, esophagus, lung, colon and head and neck [7]–[13]. The most investigated SNP in the CCND1 gene is CCND1 G870A which is located at codon 242 (nucleotide 870) in the splice donor region of exon 4 [14]. The G870 allele creates an optimal splice donor site, expressing the well-described transcript of cyclin D1 (‘transcript a’); while the A870 allele hinders the splicing event, producing a variant splice of cyclin D1 (‘transcript b’) [15]. Cyclin D1b is a more stable, constitutively nuclear protein, harboring increased transforming capability (as compared to cyclin D1a) [16], [17]. Most studies link the A-allele to increased cancer risk. However, results are inconsistent as some studies implicate the G-allele with increased cancer risk [15], [18].

Published studies have also evaluated the association between CCND1 G870A polymorphism and NPC susceptibility [19]–[23]. However, these studies showed inconsistent results. To further investigate this potential association, we firstly performed a hospital-based case-control study to evaluate the association of NPC risk with CCND1 G870A polymorphism in the central-south Chinese population, and then conducted a meta-analysis of eligible studies to obtain a more precise estimation of this association.

## Materials and Methods

### Study population

This hospital-based case-control study included 165 nasopharyngeal carcinoma (NPC) cases and 191 cancer-free individuals consecutively recruiting from Hunan Tumor Hospital between April 2011 and October 2011. The patients were diagnosed via histopathological evidence and received no treatment before the blood drawing. There were no age, sex, and stage restriction for cases. The selection criteria for controls included no family history of nasopharyngeal carcinoma and frequency matched to cases on age and sex. All recruited subjects were unrelated ethnic Chinese adults, who were resident in Changsha City (Changsha, China) or the surrounding regions. Written informed consent was obtained from all participants for the use of their blood samples in the present study. This study was approved by the institutional review board of Hunan Tumor Hospital.

### DNA extraction and genotyping assay

Genomic DNA was extracted from peripheral blood samples with standard procedures (TIANGEN BIOTECH, Beijing, China) and DNA samples were frozen at −80°C. Genotyping of CCND1 G870A polymorphism was carried out using the polymerase chain reaction-restriction fragment length polymorphism (PCR-RFLP) method. The PCR mixture consisted of 5 pmol of each primer, 1X GoTaq Master Mix (Promega Corporation, Madison, WI, USA) and 2.0 µl extracted DNA at a total volume of 15 µl. The PCR procedure included an initial melting step at 95°C for 1 min, then 35 cycles at 55°C for 1 min, at 72°C for 1 min, and a final extension step at 72°C for 10 min. The primers for PCR amplification were as follows: forward, 5′-GTG AAG TTC ATT TCC AAT CCG C-3’ and reverse, 5′-GGG ACA TCA CCC TCA CCC TCA CTT AC-3’. PCR products (15 µL) were digested with 1 U *ScrF1* at 37°C for 4 hours and visualized by electrophoresis on 3% agarose gels containing 0.5 µg/mL ethidium bromide. The PCR product of 167 base pairs (bp) (the AA genotype) was digested into fragments of 145 and 22 bp for GG and into fragments of 167, 145 and 22 bp for AG. PCR products were randomly selected for DNA sequencing validation.

### Statistical analysis

The Hardy-Weinberg equilibrium was used to test the genotype frequencies of CCND1 G870A polymorphism among the control groups. Chi-square test (χ2) was used to measure the difference in the distribution of genotype and allele frequencies between patients and controls. Odds ratios (ORs) with 95% confidence intervals (95% CIs) for the risk of NPC were calculated to estimate the relative risk using the multivariate logistic regression analysis adjusted by age and gender. Statistical analyses were performed with SPSS 19.0 software. All statistical analyses were two-sided and differences were considered to be statistically significant when *p*<0.05.

### Meta-analysis

Literature searches of the PubMed, Embase, Chinese National Knowledge Infrastructure (CNKI), China Biological Medicine Database and Wanfang Database (up to April 2014) were performed to identify eligible studies. The publication language was restricted to English and Chinese and the combination terms were “CCND1”, “cyclin D1”, “polymorphism”, “mutation or variant” and “nasopharyngeal.” The reference lists of identified studies were manually checked to include other potentially eligible trials. All eligible studies in this meta-analysis met the following criteria: case-control studies in design; investigating the association between CCND1 G870A polymorphism and NPC risk; with genotype distribution data to calculate combined ORs and 95% CIs. The major exclusion criteria were: no control population; abstract, comment, and review; duplicate of earlier publication and no usable genotype frequency data. The following data was extracted from eligible studies: first author, year of publication, country and ethnicity of study population, numbers of cases and controls, and genotype frequency of cases and controls.

The pooled odds ratio (OR) with 95% confidence interval (CI) was used to evaluate CCND1 G870A polymorphisms and NPC risk. The Q-test and *I^2^* test were used to assess the heterogeneity between studies. *I^2^*<25% indicated low heterogeneity, 25%≤*I^2^*≤50% indicated moderate heterogeneity, and *I^2^*>50% indicated large heterogeneity. If *P_Q_*<0.10 or *I^2^*>50%, the random-effects model (the DerSimonian and Laird method) was used to calculate the pooled OR. Otherwise, the fixed-effects model (Mantel-Haenszel) was selected. The significance of the pooled ORs was assessed via Z-test. The allele model (A vs. G), the co-dominant model (AA vs. GG), the dominant model (AA/AG vs. GG) and the recessive model (AA vs. AG/GG) was performed respectively. Subgroup analysis by ethnicity was also performed. Begg’s funnel plot and Egger’ linear regression test were used to assess publication bias. Hardy-Weinberg equilibrium (HWE) was test in the control groups. In this meta-analysis, all statistical analyses were performed using the software Review Manager (version 5.0) and STATA software (version 12.0).

## Results

### Characteristics of patients with NPC and healthy controls in the case-control study

A total of 356 subjects were included in this case-control study, including 165 patients with NPC and 191 healthy controls. The baseline characteristics were listed in **Table 1** **.** There were no significant differences between the groups in their gender and age.

**Table 1:** Characteristics of NPC Patients and Controls.

Characteristics	Patients	Controls	P-value
Mean age (mean ± s.d.)	46.45±11.3	44.3±10.4	0.271
Age			
<45	79(47.9)	90(47.1)	0.916
≥45	86(52.1)	101(52.9)	
Gender			
Male	118(71.5)	126(66.0)	0.261
Female	47(28.5)	65(34.0)	
T stage			
T1+T2	56(35.2)	―	
T3+T4	103(64.8)	―	
N stage			
N0	9(5.7)	―	
N1+N2+N3	150(94.3)	―	
Clinical stage			
I+II	11(6.8)	―	
III+IV	150(93.2)	―	
Metastasis			
No	146(93.0)	―	
Yes	11(7.0)	―	

Abbreviation: s.d., standard deviation.

The sum of various characteristics does not equal because of the unavailable data.

### Genotype distribution and association analysis between CCND1 G870A polymorphism and risk of NPC in the case-control study

The genotype and allele frequency distributions for G870A among the cases and controls and their associations with risk for cervical cancer are shown in **Table 2**. The frequencies of genotype GG, AG and AA of CCND1 were 7.2, 55.8 and 37.0%, respectively, in the patient group; and 16.7, 46.1 and 37.2%, respectively, in the control group. The allele frequencies for G and A were 35.2 and 64.8%, respectively, in the patient group; and 39.8 and 60.2%, respectively, in the control group. The genotype distribution was in the Hardy-Weinberg equilibrium in control group (p = 0.650). The CCND1 G870A polymorphism showed significant difference between NPC patients and healthy controls in genotype comparison (AA vs. GG: OR = 2.291, 95% CI 1.086–4.833, p = 0.030; AG vs. GG: OR = 2.788, 95% CI 0.350–5.756, p = 0.006; AA/AG vs. GG: OR = 2.566, 95% CI 1.275–5.165, p = 0.008; AA vs. AG/GG: OR = 0.991, 95% CI 0.644–1.526, p = 0.968,**Table 2**). Similar significant differences were found in age-adjusted logistic regression analysis (AA vs. GG: OR = 2.300, 95% CI 1.089–4.857, p = 0.029; AG vs. GG: OR = 2.832, 95% CI 1.367–5.867, p = 0.005; AA/AG vs. GG: OR = 2.597, 95% CI 1.288–5.237, p = 0.008; AA vs. AG/GG: OR = 0.984, 95% CI 0.638–1.518, p = 0.944, **Table 2**). However, no significant association was found between NPC patients and healthy controls in allele comparison (p = 0.203, **Table 2**).

**Table 2:** Genotype and Allele Distribution of cylin D1(CCND1) in Patients and Controls.

Polymorphism	Patient	Control	P-value^a^	Crude OR(95% CI)	P-value	Adjusted OR(95% CI)	P-value^b^
Genotype							
GG	12(7.2)	32(16.7)	0.018				
AG	92(55.8)	88(46.1)					
AA	61(37.0)	71(37.2)					
AA versus GG			0.027	2.291(1.086–4.833)	0.030	2.300(1.089–4.857)	0.029
AG versus GG			0.005	2.788(0.350–5.756)	0.006	2.832(1.367–5.867)	0.005
AA/AG versus GG			0.007	2.566(1.275–5.165)	0.008	2.597(1.288–5.237)	0.008
AA versus AG/GG			0.968	0.991(0.644–1.526)	0.968	0.984(0.638–1.518)	0.944
Allele							
G	116(35.2)	152(39.8)	0.203				
A	214(64.8)	230(60.2)					

Abbreviations: CI, confident interval; OR, odd ratio.

a P-values were calculated from two-sided chi-square tests.

b Data were calculated by logistic regression with adjustment for age and gender.

### Meta-analysis results

**Figure 1:**
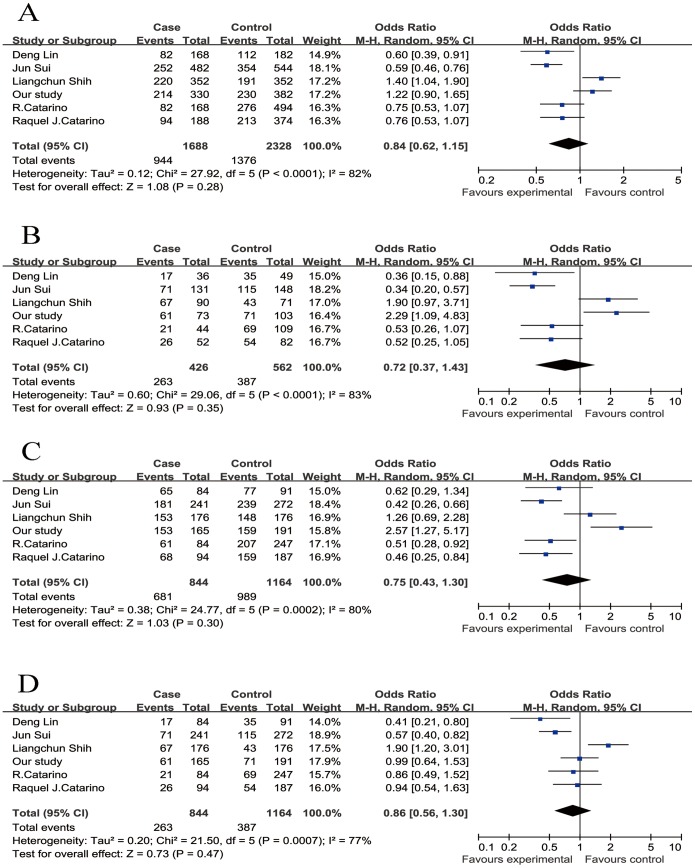
Forest plot of the risk of nasopharyngeal carcinoma associated with the CCND1 G870A polymorphism. A, the alleles model (A vs. G); B, the co-dominant model (AA vs. GG); C, the dominant model (AA/AG vs. GG); D, the recessive model (AA vs. AG/GG). Error bars indicate 95% CI. Solid squares represent each study in the meta-analysis. Solid diamonds represent pooled OR.

**Figure 2:**
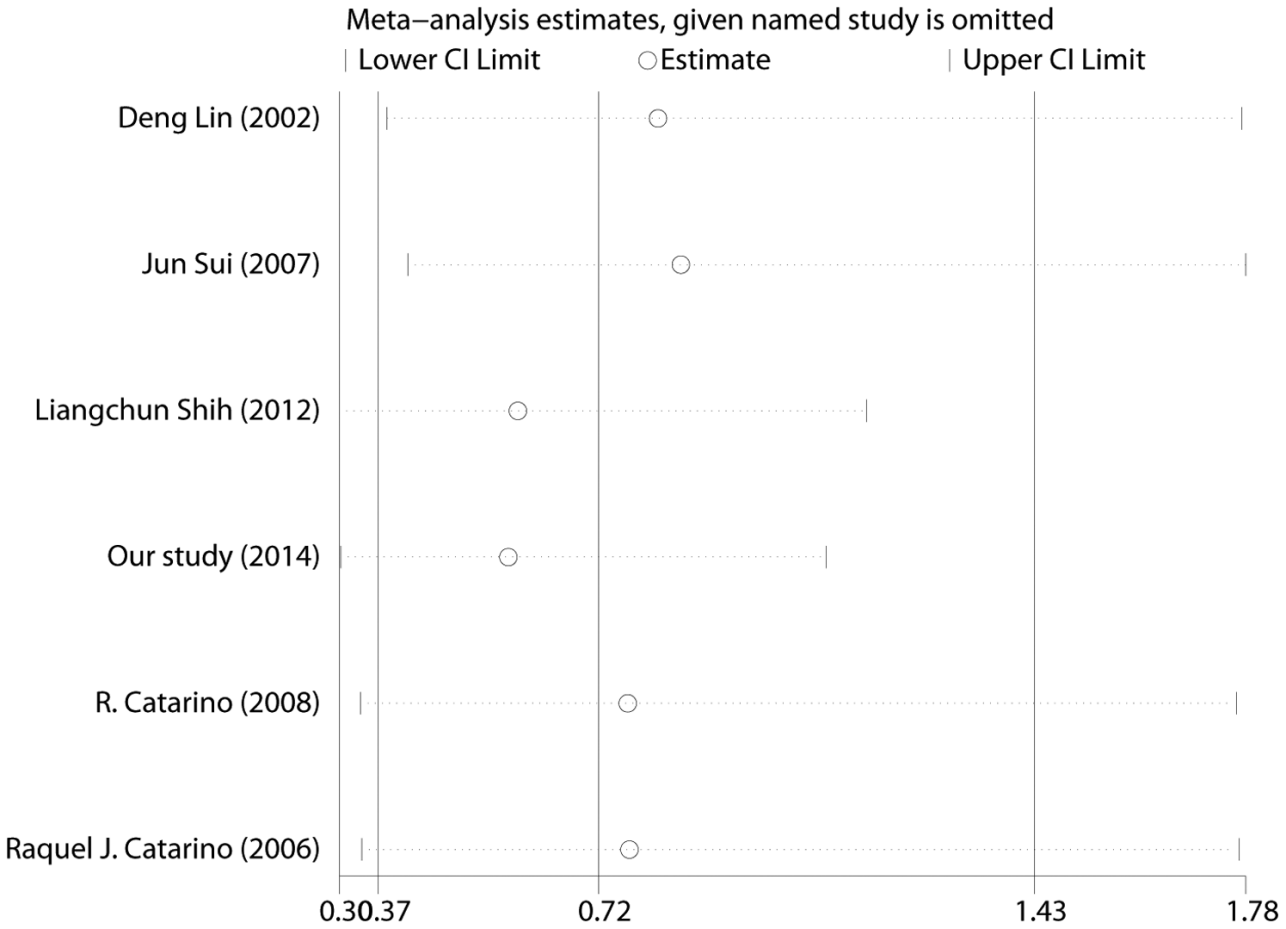
Influence analysis for the co-dominant model (AA vs. GG) in the overall meta-analysis. This figure shows the influence of individual studies on the summary OR. The middle vertical axis indicates the overall OR and the two vertical axes indicate its 95% CI. Every hollow round indicates the pooled OR when the left study is omitted in this meta-analysis. The two ends of the dotted lines represent the 95% CI.

In total, six studies (844 cases and 1164 controls) published from 2002 to 2014, including our study, were identified to be eligible studies. The detailed process of selecting and excluding articles is presented in **Figure S1.** The characteristics of all the enrolled studies that were performed in the meta-analysis were listed in **Table 3**. The genotype distributions of all studies in the control groups conformed to the HWE equilibrium except the report by Shih et al. Studies with controls not in Hardy-Weinberg equilibrium (HWE) were also considered for the meta-analysis, but they were excluded in the sensitivity analysis. Overall, the combined results indicated that CCND1 G870A polymorphism was not significantly associated with NPC risk in all genetic models (for the allele model A vs. G: OR = 0.84, 95% CI 0.62–1.15, p = 0.28; for the co-dominant model AA vs. GG: OR = 0.72, 95% CI 0.37–1.43, p = 0.35; for the dominant model AA/AG vs. GG: OR = 0.75, 95% CI 0.43–1.30, p = 0.30; for the recessive model AA vs. AG/GG: OR = 0.86, 95% CI 0.56–1.30, p = 0.47, **Fig. 1**). The sensitivity analysis indicated omission of any single study did not have significant impact on the combined ORs (**Fig. 2**). Subgroup analyses by ethnicity further showed that there was also no significant association between CCND1 G870A polymorphism and NPC risk in Asians (for the allele model A vs. G: OR = 0.89, 95% CI 0.56–1.41, p = 0.61; for the co-dominant model AA vs. GG: OR = 0.85, 95% CI 0.30–2.43, p = 0.77; for the dominant model AA/AG vs. GG: OR = 0.94, 95% CI 0.42–2.13, p = 0.89; for the recessive model AA vs. AG/GG: OR = 0.83, 95% CI 0.45–1.55, p = 0.56) (**Table 4**). However, significant associations were found in Caucasians in three genetic model (for the allele model A vs. G: OR = 0.75, 95% CI 0.59–0.97, p = 0.03; for the co-dominant model AA vs. GG: OR = 0.52, 95% CI 0.32–0.86, p = 0.01; for the dominant model AA/AG vs. GG: OR = 0.49, 95% CI 0.32–0.74, p<0. 01; for the recessive model AA vs. AG/GG: OR = 0.90, 95% CI 0.61–1.34, p = 0.60) (**Table 4**).

**Table 3:** The characteristics of the included studies in the meta analysis.

	Year	Area	Ethnicity	Genotypingmethod	No. ofcases	No. ofcontrols	Cases			Controls			HWE(Control)
First Author(ref.)							AA	AG	GG	AA	AG	GG	p
Deng Lin	2002	China	Asian	DHPLC	84	91	17(20.2)	48(57.2)	19(22.6)	35(38.5)	42(46.1)	14(15.4)	0.826
Jun Sui	2009	China	Asian	PCR-RFLP	241	272	71(29.5)	110(45.6)	60(24.9)	115(42.3)	124(45.6)	33(12.1)	1.000
Liangchun Shih	2012	Taiwan	Asian	PCR-RFLP	176	176	67(38.1)	86(48.9)	23(13.0)	43(24.4)	105(59.7)	28(15.9)	0.010
Our study	2014	China	Asian	PCR-RFLP	165	191	61(37.0)	92(55.8)	12(7.2)	71(37.2)	88(46.1)	32(16.7)	0.650
R. Catarino	2008	Portugal	European	PCR-RFLP	84	247	21(25.0)	40(47.6)	23(27.4)	69(27.9)	138(55.9)	40(16.2)	0.052
Raquel J. Catarino	2006	Portugal	European	PCR-RFLP	94	187	26(27.7)	42(44.7)	26(27.6)	54(18.9)	105(56.1)	28(15.0)	0.054

**Table 4:** Meta-analysis of cylin D1(CCND1) polymorphism and risk of NPC.

Genetic comparison	Begger’s test(z, p)	Egger’s test(t, p)	OR(95%CI)	P_OR_	I^2^ (%)	Effect model
Overall (6)						
A vs. G	0.00, 1.000	−0.26, 0.807	0.84(0.62,1.15)	0.28	0.82	R
AA vs. GG	0.00, 1.000	−0.80, 0.468	0.72(0.37,1.43)	0.35	0.83	R
AA/AG vs. GG	1.13, 0.260	−1.57, 0.193	0.75(0.43,1.30)	0.30	0.80	R
AA vs. AG/GG	0.38, 0.707	−0.00, 0.999	0.86(0.56,1.30)	0.47	0.77	R
Asians (4)						
A vs. G			0.89(0.56,1.41)	0.61	0.89	R
AA vs. GG			0.85(0.30,2.43)	0.77	0.89	R
AA/AG vs. GG			0.94(0.42,2.13)	0.89	0.86	R
AA vs. AG/GG			0.83(0.45,1.55)	0.56	0.86	R
Caucasians (2)						
A vs. G			0.75(0.59,0.97)	0.03	0.03	F
AA vs. GG			0.52(0.32,0.86)	0.01	0.00	F
AA/AG vs. GG			0.49(0.32,0.74)	0.00	0.00	F
AA vs. AG/GG			0.90(0.61,1.34)	0.60	0.00	F

Abbreviations: CI, confident interval; OR, odd ratio; R, random model; F, fixed model.

### Publication bias

**Figure 3:**
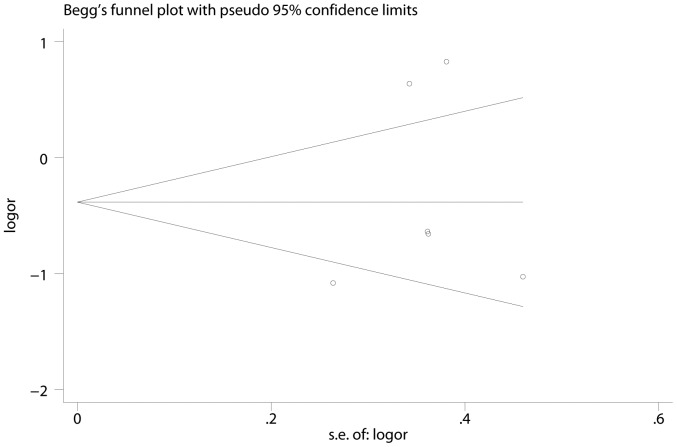
Begger’s funnel plots for publication bias of the meta-analysis on the association between CCND1 G870A polymorphism and NPC risk in co-dominant model (AA vs. GG).

The Begg’s funnel plot and Egger’s test were used to estimate the publication bias of the meta-analysis. As the shape of the funnel plots of the all genetic models seem symmetrical, this indicated low publication bias in this meta-analysis (**Fig. 3**).

## Discussion

Nasopharyngeal carcinoma is an epithelial malignancy with an unusual ethnic and geographic disparity [24]. The formation of NPC results from complex interactions between genetic backgrounds and environmental factors [25], [26]. CCND1, a key regulator of cell cycle progression, plays an important role in the G_1_/S checkpoint of the cell cycle [27]. The disorder of CCND1 would contribute to cancer development. The CCND1 G870A polymorphism will change the spliced transcript of CCND1 and lead to over expression of CCND1, which may lead to abnormal cell proliferation and contribute to cancer development. Several meta-analyses have revealed the associations between CCND1 G870A polymorphism and cancer risk, including lung cancer, oral cancer, esophageal cancer and breast cancer [28]–[30].

In recent years, the association between CCND1 G870A polymorphism and NPC risk was also investigated in several studies. The association between CCND1 polymorphisms and NPC risk was firstly reported by Deng et al., which maintained the GG and AG genotypes in NPC patients were significantly higher than those in normal controls [22]. However, inconsistent conclusions were revealed by other studies [19]–[21], [23]. Three of the studies were conducted in Asians from high NPC incidence endemic area; two were in Portugal population from the midterm incidence area of Europe. Because of the relatively small sample size and different patient population, studies on the association of CCND1 polymorphisms and NPC risk showed inconsistent results. Therefore, a case-control study of the central-south Chinese population, along with a meta-analysis on NPC, was performed to provide the most comprehensive evaluation of the association between CCND1 G870A polymorphism and NPC risk. The results of our case-control study showed that the A allele of the CCND1 G870A polymorphism might be associated with nasopharyngeal carcinoma (NPC) in the Chinese population. However, the further meta-analysis showed that the CCND1 G870A polymorphism was not associated with NPC risk in overall population but significant in Caucasians. This result indicated that the association between CCND1 G870A polymorphism and NPC risk was various in different ethnicities.

There was a published meta-analysis on CCND1 G870A polymorphism and NPC risk [31]. The study suggested that there was no significant association between G870A polymorphism and risk of NPC. In contrast with the previous study, there were some advantages in our updated meta-analysis: firstly, the sample size was larger in our study. Our meta-analysis included our recent case-control study. A total of six case-control studies with 844 cases and 1164 controls give a greater power to evaluate the association. Secondly, the previous study showed that there were three studies which the genotype frequencies in the controls significantly deviated from the HWE. However, the extracted data showed that there was at least one of the group data less than 40; therefore Fisher’s exact test was more suitable to evaluate the HWE. We found that there was only one study deviated from the HWE using the Fisher’s exact test which seem to contradict with the previous study. Thirdly, sensitivity analysis was performed by excluding some case-control studies in our meta-analysis. The exclusion of the study departing from HWE would not have changed our results. Moreover, exclusion of any single study did not alter the pooled results. The sensitivity analysis added robustness to our finding.

In the present meta-analysis, significant heterogeneity was observed for the association between CCND1 G870A polymorphism and NPC risk among the six studies. By performing subgroup analysis, we found the the *I^2^* values were less than 50% and *P_Q_* were greater than 0.1 in the Caucasians. However, heterogeneity was still present in the Asians group. The potential factors that may account for the heterogeneity included study design and sample population. Among the four study in the Asians group, the study by Jun Sui et al, Liangchun Shih et al, and our study were designed as a hospital-based case-control study and recruited aged-match controls; whereas the study by Deng Lin et al was not a stringent hospital-based study. The cases of the study by Deng Lin et al were recruited from three hospitals in a city and the controls with lower age were recruited from a university in the same city. The histological types were different in the four studies. All cases of the study by Jun Sui et al were diagnosed as squamous cell carcinomas; most cases (82/84) of the study by Deng Lin et al were poorly differentiated squamous cell carcinomas; whereas histological types of the cases in the study by Jun Sui et al and our study were without restriction. Nevertheless, we are unable to determine the precise factors that account for the heterogeneity due to the limited data.

There are some limitations in this study. Firstly, the sample sizes of cases and controls were relatively small, especially in the subgroup of Caucasians. There were only two studies on Caucasians and it might result in the false positive findings in our meta-analysis. All results should be interpreted with very caution. Secondly, due to the limited data, we did not carry out subgroup analysis to other factors, which may participate in the progression of disease, such as gene-gene, smoking and other lifestyle. Thirdly, obvious heterogeneity was observed in our meta-analysis. The populations, ethnicity, habits, geographical location and study designs may contribute to the heterogeneity.

In conclusion, our case-control study suggested that there was a significant association between the CCND1 G870A polymorphism and NPC risk in the Chinese people. Furthermore, our meta-analysis showed that CCND1 G870A polymorphism was at significantly greater risk for NPC in the Caucasian population. However, these findings should be validated by more studies with large sample sizes, gene-gene, gene-environment interactions, well-designs and more diverse ethnic groups’ data.

## Supporting Information

Figure S1 **Flow chart of study selection by using electronic database.**
(PPT)

Checklist S1 **PRISMA 2009 Checklist.**
(DOC)
