# Supplementary material for: Cyclin D1 G870A Polymorphism and Risk of Nasopharyngeal Carcinoma: A Case-Control Study and Meta-Analysis
Source: PLoS One. 2014 Nov 19;9(11):e113299. doi: 10.1371/journal.pone.0113299 (PMC4237450; doi:10.1371/journal.pone.0113299)
Supplement: Figure S1 — Flow chart of study selection by using electronic database. (PPT) [file pone.0113299.s001.ppt]

## Slide 1
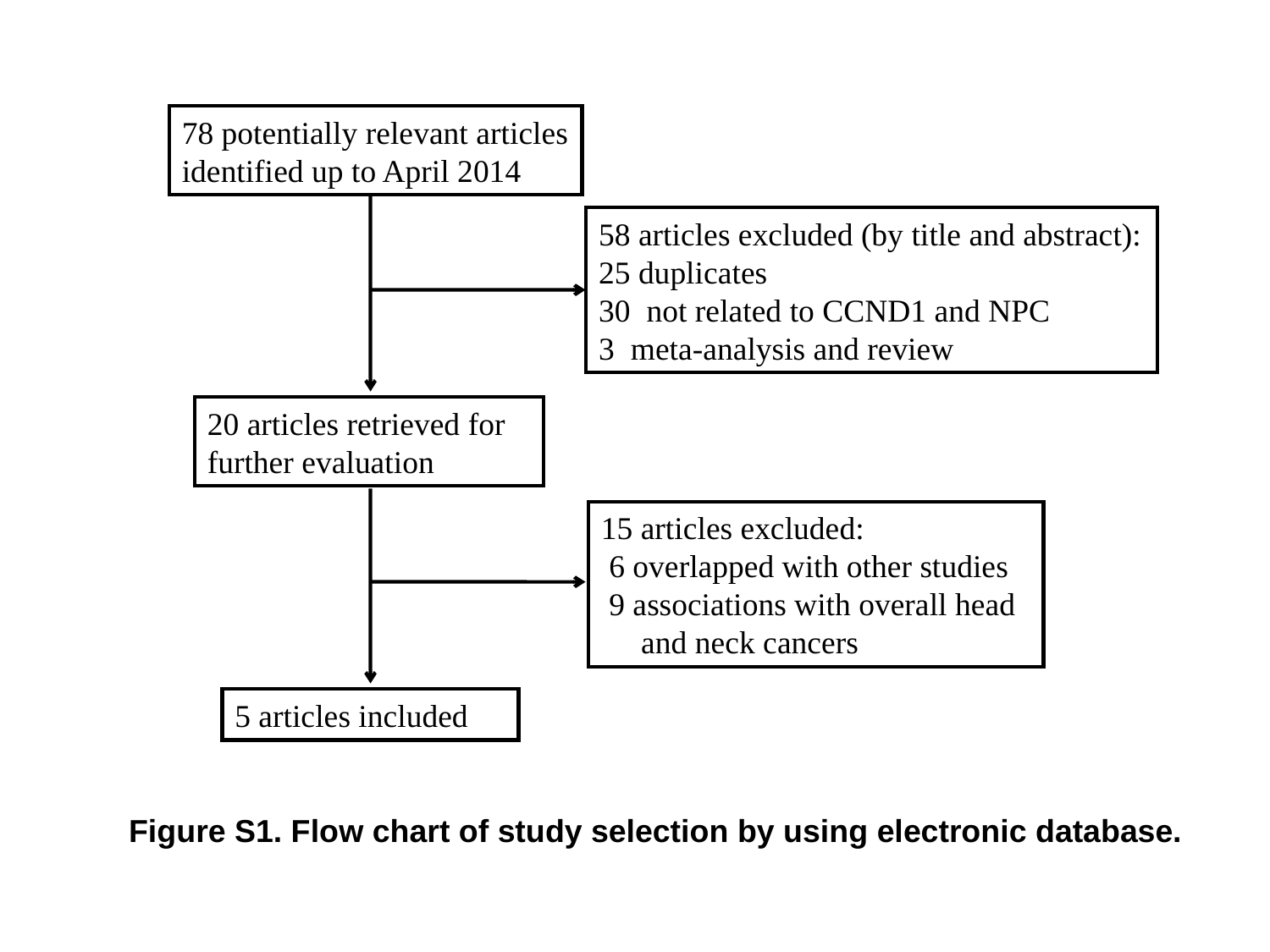

78 potentially relevant articles identified up to April 2014
58 articles excluded (by title and abstract):
25 duplicates
30 not related to CCND1 and NPC
3 meta-analysis and review
20 articles retrieved for further evaluation
15 articles excluded:
 6 overlapped with other studies
 9 associations with overall head and neck cancers
5 articles included
Figure S1. Flow chart of study selection by using electronic database.
